# Association of the neonatal sequential organ failure assessment score with neurological outcomes in infants diagnosed with hypoxic-ischemic encephalopathy

**DOI:** 10.3389/fped.2026.1844058

**Published:** 2026-05-29

**Authors:** Kaitlyn Lagnese, Shamil Sheth, Shannon Vice, Dhanashree Rajderkar, Juan C. Roig, Michael Weiss, James L. Wynn

**Affiliations:** 1 Department of Pediatrics, Division of Neonatology, University of Florida, Gainesville, FL, United States; 2 Radiology, Texas Children's Hospital and Baylor College of Medicine, Houston, TX, United States

**Keywords:** Bayley, HIE, NDI, neurodevelopmental outcomes, NICu, nSOFA, therapeutic hypothermia, weeke score

## Abstract

**Introduction:**

Early identification of neurodevelopmental impairment (NDI) risk in neonates with hypoxic-ischemic encephalopathy (HIE) treated with therapeutic hypothermia (TH) may enable more precise care and improved outcomes.

**Methods:**

This was an exploratory, single-center, retrospective cohort study that included inborn infants admitted from January 2012– March 2023 with a diagnosis of HIE, who received TH within 6 hours of life and underwent a Weeke-scored brain MRI. Hourly neonatal sequential organ failure assessment (nSOFA) scores and laboratory maximum and minimum values were calculated using raw data.

**Results:**

Among 122 infants, 36 (29.5%) had an abnormal MRI (Weeke score ≥5). Bayley-III testing performed at ≥12 months of age was available for 66 infants, of whom 23 (34.8%) met criteria for NDI (any domain composite score <85). Models using laboratory values and the maximum nSOFA yielded good predictive accuracy for both an abnormal Weeke score (AUROC 0.79, 95% CI 0.70–0.88; NPV 81%, PPV 74%) and NDI (AUROC 0.78, 95% CI 0.66–0.89; NPV 78%, PPV 75%).

**Discussion:**

Early physiologic and laboratory measures predicted both MRI-defined injury and NDI, suggesting that early illness severity reflects underlying injury processes across outcome domains. Integration of these measures may support more precise risk stratification and prognostic assessment in infants with HIE.

## Introduction

Hypoxic-ischemic encephalopathy (HIE) remains a major cause of death and long-term disabilities including motor, sensory, and cognitive impairment (1). Therapeutic hypothermia (TH) is standard of care for infants diagnosed with moderate to severe HIE (2). Despite TH, some infants with HIE will exhibit poor long-term neurologic outcomes across a spectrum of severity including neurodevelopmental impairment (NDI) (3–6). Progress has been made in the detection of death or severe impairment (7, 8) and outcome uncertainty remains for a majority of HIE patients even with the availability of magnetic resonance imaging (MRI). Management of this uncertainty is challenging for both parents and clinicians and underscores the need for clinically useful NDI prediction models in this population.

Newborns with HIE often exhibit a spectrum of organ dysfunction (9). Life-threating neonatal organ dysfunction in the respiratory, cardiovascular, and hematologic systems can be quantified by the neonatal Sequential Organ Failure Assessment (nSOFA) (10). Validated in multicenter studies, the nSOFA has strong utility to predict all-cause mortality (11) including ELBWs (12), as well as disease-specific mortality across sepsis and necrotizing enterocolitis (13–15). Kurul et al. showed the cumulative nSOFA score during late onset sepsis events in preterm infants was positively associated with neurodevelopmental outcomes at two years of age (16). The relationship between early nSOFA scores and poor neurologic outcomes among HIE patients is unclear. Similarly, laboratory markers of injury and inflammation may possess utility in predicting the severity of encephalopathy and future NDI in infants with HIE. While a single biomarker is unlikely to reliably predict long-term outcomes (17), the combination of early serial measures of organ dysfunction alongside multiple laboratory markers of injury and inflammation may be more informative. *This study was designed as an exploratory analysis to evaluate early physiologic and laboratory markers associated with MRI-defined brain injury and neurodevelopmental outcomes, rather than to develop or validate a clinical prediction model.* We hypothesized that a greater extent of early organ dysfunction and laboratory abnormalities would be strongly associated with both brain injury on MRI and NDI. We determined the relationship between early physiologic and laboratory measures and neurologic outcomes in newborns with HIE treated with therapeutic hypothermia.

## Materials and methods

### Study design and approval

This single-center retrospective cohort study was approved by the University of Florida Institutional Review Board (*IRB 201902780*). We included inborn infants admitted to the UF Neonatal Intensive Care Unit between January 2012 and April 2023 who were diagnosed with HIE and received TH.

### Inclusion and exclusion criteria

All infants were diagnosed with HIE and met eligibility criteria for whole-body TH, which is standardized according to institutional protocol. The entry criteria for hypothermia was a gestational age of ≥35 weeks, birth weight of ≥1.8 kg, and ≤6 h of life (HOL) at the time of evaluation. Encephalopathy was the presence of seizures or abnormalities on a modified Sarnat examination, which assessed level of consciousness, spontaneous activity, posture and tone, primitive reflexes (suck and Moro), and autonomic function (including pupils, heart rate, and respirations) (4, 18). Hypoxic-ischemic injury was one or more of the following criteria: arterial pH: ≤7.0 and/or base deficit ≥16 mmol/L, arterial pH: 7.01–7.15 and/or base deficit 10–15.9 mmol/L, or an acute perinatal event including cord prolapse, fetal heart rate decelerations, or uterine rupture. Exclusion criteria included the presence of major congenital anomalies, known chromosomal abnormalities, lack of a Weeke-scored MRI, initiation of TH beyond 6 HOL and outborn neonates. TH was administered using the CritiCool™ blanket device (Mennen Medical Corp., Feasterville–Trevose, PA) with a target temperature of 33.5 °C for 72 h*.* For the purposes of this study, the initial Sarnat examination performed to determine eligibility for TH was used.

### Data collection

An integrated data repository was created with all clinical data in the electronic health record (EHR) for all HIE patients. All data were extracted from the IDR or via chart review. Modified Sarnat scores and times were manually extracted for each patient.

### Neonatal sequential organ failure assessment (nSOFA)

The nSOFA score ranges from 0 to 15 and is calculated using the presence of mechanical ventilation and SpO2/FiO2 ratio for respiratory dysfunction, presence of vasoactive medications and/or corticosteroids for cardiovascular dysfunction, and platelet count for hematologic dysfunction (Table 1) (10). We calculated q1 h nSOFA scores using raw data from the IDR with random audits to ensure score accuracy. To permit hourly calculations for the hematologic nSOFA subscore based on laboratory values (platelet), a last-one-copied-forward approach was used until a new value was available. When calculation of >1 nSOFA score was possible within a single hour for a single patient, the maximum nSOFA score based on simultaneously calculated nSOFA components was used. The nSOFA index was the sum of nSOFA scores divided by the number of hours scored over a specific interval, and the maximum nSOFA score was the greatest nSOFA score over a specific interval.

**Table 1:** Neonatal sequential organ failure assessment (nSOFA) scoring system (0-15 points).

Respiratory score	0	2	4	6	8
Criteria	Not intubated ***OR*** Intubated, SpO_2_/FiO_2_ ≥ 300	Intubated, SpO_2_/FiO_2_ <300	Intubated, SpO_2_/FiO_2_ <200	Intubated, SpO_2_/FiO_2_ <150	Intubated, SpO_2_/FiO_2_ <100

Cardiovascular score	0	1	2	3	4
Criteria	No inotropes ***AND*** No systemic steroids	No inotropes ***AND*** Systemic steroid treatment	One inotrope ***AND*** No systemic steroids	Two or more inotropes ***OR*** One inotrope AND systemic steroid treatment	Two or more inotropes ***AND*** Systemic steroid treatment

Hematologic score	0	1	2	3
Criteria	Platelet count* ≥150 × 10^3^	Platelet count 100–149 × 10^3^	Platelet count <100 × 10^3^	Platelet count <50 × 10^3^

* Most current value.

### Laboratory analyses

Within the first 12 h of life, the following labs were obtained on admission per protocol: cord gas, blood gas and lactate, complete blood count, electrolytes, hepatic function panel, coagulation studies. Blood gas and lactate were then repeated every 6 h, or more frequently as needed for ventilation management. Maximum and minimum values for each parameter from birth to 12 HOL were identified.

### MRI scoring

MRI scans were performed on either a Siemens Magneto Verio 3 T or Siemens Magnetom Avanto 1.5 T scanner after TH was completed. A single blinded subspecialty board-certified neuroradiologist with over 10 years of experience in neonatal imaging interpreted all the MRI images using the Weeke scoring system. The Weeke scoring system evaluates brain injury across three regions: deep gray matter, white matter/cortex, and the cerebellum, with an additional subscore assessing the presence of intraventricular hemorrhage, subdural hemorrhage, and cerebral sinovenous thrombosis (19). Each anatomical region is systematically scored based on the extent and distribution of injury. The total score is calculated by summing the scores from the gray matter, white matter/cortex, cerebellum, and additional categories, with a maximum score of 55. If 1H-MRS data are available, abnormalities in the basal ganglia and thalamus, such as reduced N-acetyl aspartate or elevated lactate peaks, are incorporated into the gray matter subscore, increasing the total score to a maximum of 57. A Weeke score of 0–4 was classified as normal, while a Weeke score of ≥5 was classified as abnormal (20).

### Neurodevelopmental testing

The Bayley Scales of Infant and Toddler Development, Third Edition (Bayley-III), were administered by a trained occupational therapist at age 12 months or older. Neurodevelopmental impairment (NDI) was defined as a Bayley-III composite score of less than 85 in any of the three domains: cognition, motor, or language.

### Statistical analysis

Tests of normality were performed on all continuous data. Parametric continuous variables were compared using student's t-test and presented as means and standard deviation (SD). Non-parametric continuous variables were compared using the Mann–Whitney *U* test and presented as medians with interquartile range (IQR). Categorical variables were compared using the Chi Square test and presented as percentages. The threshold for significance was set to a *p* < 0.05. Values with the greatest significance were used in a multivariate logistic regression (MVLR) model and subsequently area under the receiver operating characteristic (AUROC) curve with Wilson-Brown method was calculated for each outcome group. Agreement between Bayley and Weeke classifications was assessed using Cohen's kappa. Cross-tabulation was used to summarize concordance and discordance between methods. When Bayley was treated as the reference standard, sensitivity, specificity, and predictive values for Weeke classification were calculated. Calculation of hourly nSOFA scores on raw data, Cohen's kappa, and probability tree creation was done using R (version 4.2.2). Analyses were performed in GraphPad Prism (version 11.0).

## Results

### Cohort demographics

A total of 9,752 infants were admitted from January 2012 through April 2023, of whom 344 underwent TH. After applying exclusion criteria, 122 infants were analyzed (52.5% male, mean gestational age 38.6 weeks (SD 1.7), mean birth weight 3,450 g (SD: 682). Bayley-III testing was completed in 66/122 (54.1%) infants, and of those tested 23 (34.8%) met criteria for NDI. Among the remaining 56 infants, 3 died and 53 were lost to follow-up after discharge. An abnormal Weeke score (≥5) was present in 36/122 (29.5%) infants. All three deaths were the result of a redirection of care due to the extent of brain injury and occurred only in infants with abnormal Weeke scores. Covariates were compared by Weeke groups (Table 2) and Bayley groups (Table 3). Compared to patients with a normal Weeke score, those with an abnormal Weeke score had higher frequency of low 5- and 10-min Apgar scores, non-reassuring fetal heart tones, Sarnat 3 assignment, antiepileptic drug use, and intubation (all *p* < 0.05). Conversely, length of stay was the only significant difference in the covariates examined between those with and without NDI. Among patients a Weeke score and a Bayley score (*n* = 66), 20 (30%) patients had an abnormal Weeke and 23 (35%) had an abnormal Bayley. Agreement between Bayley and Weeke classifications was 62%, with discordance in 38% of cases. Agreement beyond chance was poor (*κ* = 0.14). Using Bayley as the reference, Weeke demonstrated low sensitivity (39%) and moderate specificity (74%).

**Table 2:** Cohort demographics by Weeke grouping.

Infant characteristics	Normal Weeke (*n* = 86)	Abnormal Weeke (*n* = 36)	*p*-value
Male, *n* (%)	45 (52%)	19 (53%)	0.96
Gestational age, weeks, median (IQR)	39 (37, 40)	39 (38, 40)	0.36
Birthweight, grams, mean ± SD	3,459 ± 705	3,427 ± 633	0.80
C-section delivery, *n* (%)	54 (62.8%)	18 (50%)	0.19
Non-reassuring fetal heart tones, *n* (%)	24 (30%)	19 (52.8%)	<0.01
Sentinel event*, *n* (%)	38 (44.2%)	11 (30.6%)	0.16
No Sentinel event*, *n* (%)	48 (55.8%)	25 (69.4%)	0.16
Apgar score 1 min, median (IQR)	2 (1, 3)	2 (1, 2)	0.34
Apgar score 5 min, mean ± SD	5 ± 2	4 ± 3	0.02
Apgar score 10 min, mean ± SD	6 ± 2	4 ± 3	<0.01
Sarnat score II, *n* (%)	52 (60%)	18 (50%)	0.29
Sarnat score III, *n* (%)	14 (16.3%)	15 (41.6%)	<0.01
Length of stay, median (IQR)	11 (8, 19)	13 (9, 36)	0.13
Intubation, *n* (%)	43 (50%)	25 (69%)	0.07
Vasoactive inotropic medications, *n* (%)	15 (17.4%)	11 (30.6%)	0.15
Antiepileptic drug use, *n* (%)	13 (15.1%)	24 (66.7%)	<0.01
Arterial cord pH, mean ± SD	7.03 ± 0.15	7.02 ± 0.20	0.93
Arterial cord base deficit, mean ± SD	−13.6 ± 6.15	−11.8 ± 4.53	0.15
Maximum nSOFA, median (IQR)	0 (0, 2)	1 (0, 8)	0.008

* Sentinel event definition includes placental abruption, cord prolapse, uterine rupture, shoulder dystocia.

**Table 3:** Cohort demographics by NDI grouping.

Infant characteristics	No NDI (*n* = 43)	NDI (*n* = 23)	*p*-value
Male, *n* (%)	21 (48.8%)	10 (43.5%)	0.68
Gestational age, weeks, median (IQR)	39 (37, 40)	39 (38, 40)	0.8
Birthweight, grams, mean ± SD	3,425 ± 671	3,429 ± 655	0.99
C-section delivery, *n* (%)	21 (48.8%)	13 (56.6%)	0.55
Non-reassuring fetal heart tones, *n* (%)	16 (37.2%)	9 (39.1%)	0.88
Sentinel event*, *n* (%)	15 (34.9%)	12 (52.2%)	0.17
No Sentinel event*, *n* (%)	28 (65.1%)	11 (47.8%)	0.17
Apgar score 1 min, median (IQR)	2 (1, 3)	2 (1, 2)	0.37
Apgar score 5 min, mean ± SD	5 ± 2	4 ± 2	0.07
Apgar score 10 min, mean ± SD	6 ± 2	5 ± 2	0.07
Sarnat score II, *n* (%)	25 (58%)	14 (61%)	0.83
Sarnat score III, *n* (%)	7 (16.3%)	7 (30.4%)	0.18
Length of stay, median (IQR)	10 (8, 18)	15 (9, 41)	0.03
Intubation, *n* (%)	22 (51.2%)	16 (69.5%)	0.09
Vasoactive inotropic medications, *n* (%)	7 (16.3%)	5 (21.7%)	0.73
Antiepileptic drug use, *n* (%)	12 (27.9%)	9 (39%)	0.35
Arterial cord pH, mean ± SD	7.01 ± 0.14	7.07 ± 0.15	0.22
Arterial cord base deficit, mean ± SD	−14.2 ± 5.20	−12.5 ± 5.38	0.32
Maximum nSOFA, median (IQR)	0 (0, 1)	2 (0, 8)	0.02

* Sentinel event definition includes placental abruption, cord prolapse, uterine rupture, shoulder dystocia.

### nSOFA scores in patients with and without abnormal Weeke or NDI

**Figure 1:**
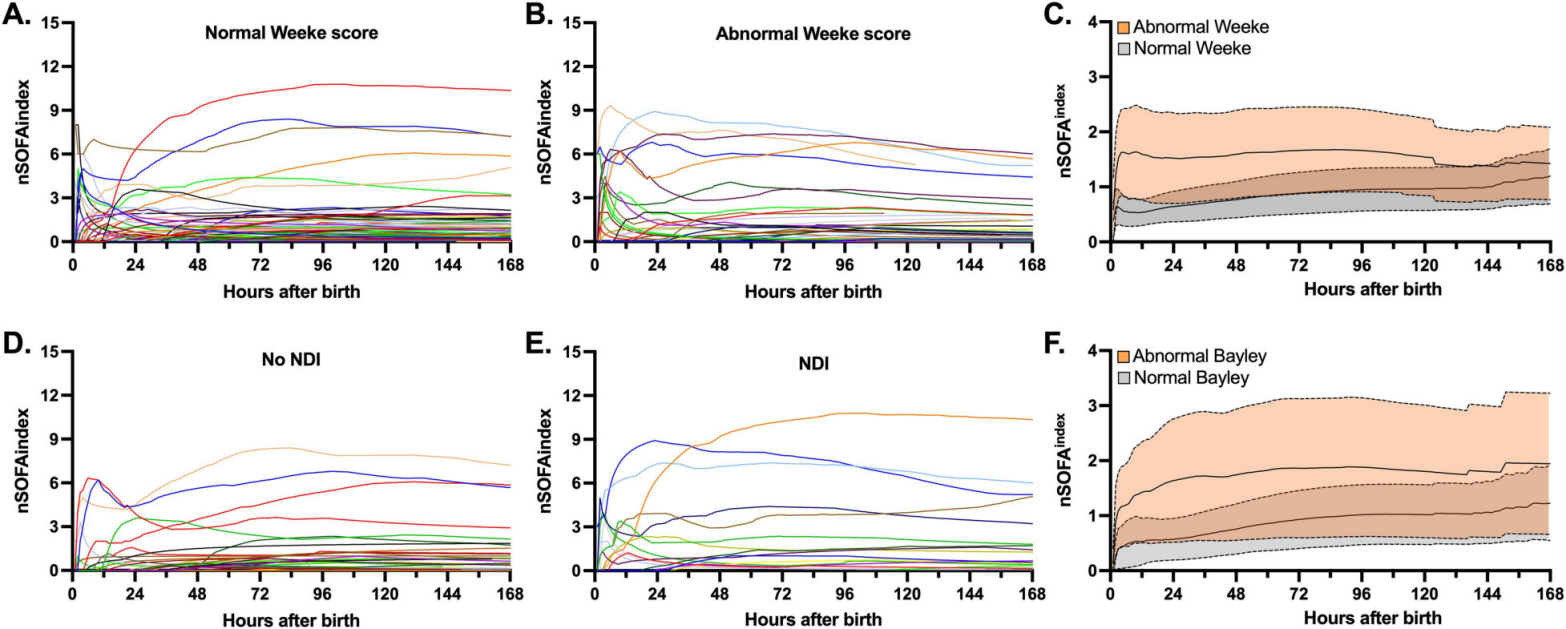
Hourly nSOFAindex profiles by Weeke and Bayley grouping. Individual patient nSOFAindex (running average score) trajectories for patients with a normal Weeke score **(A)** or abnormal Weeke score **(B)**. **(C)** Hourly nSOFAindex score mean (internal line) and 95% confidence intervals (surrounding band) for the first 168 h of life by Weeke score outcome group. Individual patient nSOFAindex trajectories for patients without NDI **(D)** and with NDI **(E)**. **(F)** Hourly nSOFAindex score mean (internal line) and 95% confidence intervals (surrounding band) for the first 168 h of life by Bayley-III score outcome group.

The maximum nSOFA score in the first 12 h after birth was greater in patient with abnormal Weeke scores (Table 2) as well as in those with NDI compared to those with a normal Weeke score or without NDI, respectively (Table 3). Although, the mean hourly values were distinct, hourly nSOFA trajectories for Weeke and Bayley groups did not exhibit separation of the 95% confidence intervals in the first 12 HOL (Figure 1).

### Laboratory values in patients with and without abnormal Weeke or NDI

Abnormal laboratory values were associated with abnormal Weeke scores or NDI (*p* < 0.01) (Supplementary Figures 1, 2). Compared to patients with normal Weeke score, measures of end organ function (creatinine and AST/ALT), acidosis (gas pH, pCO2, base deficit, lactate, serum CO2), coagulopathy (PT/INR, platelets), and metabolism (glucose) were statistically different in patients with an abnormal Weeke score. Similarly, compared to patients without NDI, measures of acidosis (gas pH, base deficit, lactate, pCO2, serum CO2, serum chloride) were statistically different in those with NDI.

### Modeling

The Sarnat score was significantly associated with worse MRI outcomes in the cohort. To examine the relationship between Sarnat score and nSOFA, we evaluated three models: Sarnat score alone, maximum nSOFA alone, and Sarnat score plus maximum nSOFA (Supplementary Table 1). The AUROC was 0.658 for Sarnat score alone, 0.633 for maximum nSOFA alone, and 0.693 for the combined model. The combined model increased the AUROC by 0.035 compared with Sarnat score alone and by 0.060 compared with maximum nSOFA alone. The integrated discrimination improvement for the combined model was 0.029 compared with Sarnat score alone and 0.036 compared with maximum nSOFA alone.

Univariate analyses were performed to identify markers of an abnormal Weeke score (Table 4). To prevent overfitting, the best model ultimately included 3 laboratory variables (maximum lactate, maximum prothrombin time, minimum blood gas base deficit) which yielded an AUROC of 0.79 (95% CI: 0.70–0.88, *p*-value <0.001). Negative predictive power was 81.1%, while positive predictive power was 73.9% (Supplementary Table 2).

**Table 4:** Univariate odd's ratios for abnormal Weeke and NDI.

Abnormal Weeke		
Covariate*	OR (95% CI)	*P*-value
Minimum blood gas base deficit	0.84 (0.77–0.91)	<0.001
Maximum prothrombin time	1.19 (1.1–1.31)	<0.001
Maximum lactate	1.16 (1.08–1.27)	<0.001
Minimum pCO2	0.85 (0.78–0.93)	<0.001
Maximum nSOFA	1.18 (1.05–1.34)	0.006
Apgar at 5 min	0.80 (0.68–0.95)	0.011
Receipt of vasoactive inotropic drug	4.46 (1.03–22.81)	0.049
Sentinel event	1.44 (0.65–3.16)	0.366

NDI		
Maximum lactate	1.22 (1.09–1.39)	<0.001
Minimum blood gas base deficit	0.88 (0.80–0.97)	0.010
Maximum nSOFA	1.22 (1.04–1.45)	0.013
Minimum pCO2	0.88 (0.78–0.98)	0.021
Receipt of vasoactive inotropic drug	8.84 (1.21–179.31)	0.031
Apgar at 5 min	0.79 (0.61–1.01)	0.058
Sentinel event	0.61 (0.19–1.96)	0.396
Maximum prothrombin time	1.01 (0.98–1.03)	0.560

* All laboratory covariates within 12 h after birth.

Univariate analyses were also performed to identify markers of NDI (Table 4). Comparison of clinical characteristics among those with and without a Bayley assessment revealed only an increased frequency of Sarnat 2 staging was present among those without (Supplementary Table 3). The best model for NDI used the minimum blood gas base deficit, maximum lactate, and maximum nSOFA score from 0 to 12 HOL with an AUROC of 0.78 (0.66–0.89, *p* < 0.001). Negative predictive power was 78.0% while positive predictive power was 75.0% (Supplementary Table 4).

Probability tree models for abnormal Weeke scores and NDI were empirically derived using combinations of covariates. To prevent overfitting, a maximum of three covariates were chosen based on impact on the probability tree and included sentinel event status, maximum lactate within 12 h after birth, and 5-min Apgar score for probability of an abnormal Weeke. Despite attempts to include 3 covariates, only maximum lactate and maximum nSOFA score had an impact on probability of NDI. For an abnormal Weeke, the initial node was a maximum lactate cutoff of 7 mmol/L followed by a 5-min Apgar score cutoff of 3 and concluding with the presence or absence of a sentinel event (Supplementary Figure 3). Infants with maximum lactate >7 in the first 12 h after birth and a 5-min Apgar ≤3 were most likely to have abnormal Weeke scores, regardless of sentinel event status (without 62%; with 67%). In contrast, 9% of infants with a maximum lactate ≤7 and a 5-min Apgar ≥3 and no sentinel event had an abnormal Weeke score.

Using maximum lactate and maximum nSOFA score, newborns with maximum lactate >12 within the first 12 HOL (initial node) had a substantially higher likelihood of NDI (>65%), independent of the maximum nSOFA score (67% when maximum nSOFA ≤1; 80% when maximum nSOFA was ≥2) (Supplementary Figure 4). Among those with a maximum lactate <12 in the first 12 HOL, probability of NDI was lower (14%) when maximum nSOFA ≤1 but increased 2.9-fold to 40% when maximum nSOFA ≥2.

## Discussion

Early laboratory and physiologic measures within 12 h after birth yielded good accuracy for both an abnormal Weeke score and NDI in patients with HIE. The limited agreement between imaging and functional outcomes suggests that these represent distinct dimensions of injury, both of which are captured by early physiologic derangements. Integrating these measures with existing tools may improve risk stratification and prognostic counseling.

Structural brain injury assessed by MRI and functional neurodevelopmental outcomes demonstrated poor agreement, with substantial discordance between Weeke and Bayley classifications. This observation is consistent with prior studies and suggests that MRI-defined injury may not fully capture long-term neurodevelopmental trajectory (21). These findings indicate that imaging-defined injury and functional neurodevelopmental outcomes represent related but distinct dimensions of neonatal brain injury expression. Notably, this discordance was observed within an exclusively inborn cohort with standardized early care, suggesting that these differences are unlikely to be explained solely by variability in early management. Instead, the persistence of discordance despite this uniformity suggests that structural brain injury and functional outcome may reflect distinct manifestations of a shared underlying hypoxic-ischemic injury process. Together, these findings support the integration of early clinical, laboratory, and physiologic data to improve precision in risk stratification and prognostic assessment.

A single laboratory value is unlikely to reliably predict long-term neurodevelopmental outcomes (17). In contrast, we found that the combination of peak organ dysfunction and laboratory values within the first 12 h after birth was strongly associated with subsequent NDI. These findings suggest that early postnatal physiology reflects underlying brain injury trajectories that precede both imaging findings and later functional assessments, providing a temporally proximal and integrated representation of injury severity during a critical window of pathophysiologic evolution. This is consistent with the concept that HIE represents a spectrum of systemic organ dysfunction, with severity of multisystem involvement contributing to neurologic outcome. Prior work has demonstrated that higher nSOFA scores are associated with increased in-hospital mortality in infants with HIE (22), and our findings extend this observation to long-term neurodevelopmental outcomes. Current predictors of neurodevelopmental impairment, including MRI and early neurologic examination findings such as suck (7), have recognized limitations, including interobserver variability and incomplete capture of outcome variability by imaging in less severe cases (21). Together, these findings support the role of early physiologic and laboratory measures as an objective and clinically accessible approach to characterizing disease severity in infants with HIE.

### Limitations

This study is limited by its retrospective design and cannot be used to inform care. Restricting the cohort to inborn patients allowed for more complete and consistent clinical data capture from birth through discharge and reduced missing data. However, this approach decreased the overall sample size and may limit generalizability to outborn populations. Another limitation was the incomplete availability of Bayley-III assessments. Infants born earlier in the study period had lower rates of outpatient follow-up at our HIE clinic, leading to missing neurodevelopmental outcome data. Although follow-up rates improved over time, social determinants of health, including transportation barriers, limited timely completion of assessments for some families. Consequently, Bayley-III testing was performed across a broader age range (12–31 months). In addition, Bayley-III has been reported to underestimate developmental impairment in some high-risk populations, which may have led to underestimation of neurodevelopmental impairment in this cohort. The limited number of NDI events remains an important limitation; however, the final model was restricted to 3 markers to reduce overfitting, and findings should be interpreted as exploratory pending external validation. The nSOFA score was evaluated as a composite measure of physiologic illness severity, consistent with its original design, and component-level analyses were not performed. Finally, although this exploratory analysis identified early physiologic and laboratory markers associated with MRI-defined brain injury and neurodevelopmental outcomes, the findings should not be interpreted as a validated clinical prediction tool. External validation in larger, independent cohorts is required to assess model performance, calibration, and generalizability before clinical application.

## Conclusion

Early laboratory and physiologic measures within 12 h after birth yielded good accuracy for both an abnormal Weeke score and NDI in patients with HIE. The discordance between imaging and functional outcomes indicates that these represent distinct dimensions of brain injury, both of which are reflected in early physiologic derangements. These findings support the use of nSOFA as a potential adjunct to established clinical and laboratory markers, rather than as a replacement, to improve early risk stratification and prognostic assessment. The added value of nSOFA appears to be greatest when incorporated into a combined early physiologic model.

## Data Availability

The datasets presented in this article are not readily available because not available due to the vulnerable population. Requests to access the datasets should be directed to Michael Weiss, weissmd@peds.ufl.edu.
