# Supplementary material for: Association of the neonatal sequential organ failure assessment score with neurological outcomes in infants diagnosed with hypoxic-ischemic encephalopathy
Source: Front Pediatr. 2026 May 29;14:1844058. doi: 10.3389/fped.2026.1844058 (PMC13260550; doi:10.3389/fped.2026.1844058)

Association of the Neonatal Sequential Organ Failure Assessment Score with Neurological Outcomes in Infants Diagnosed with Hypoxic-Ischemic Encephalopathy

Kaitlyn Lagnese[1], Shamil Sheth[1], Shannon Vice[1], Dhanashree Rajderkar[2], Juan C Roig[1], Michael Weiss[1*], James L. Wynn[1*]

*-contributed equally

[1]Department of Pediatrics, Division of Neonatology, University of Florida, Gainesville, FL, USA

[2]Department of Radiology, Division of Pediatric Radiology, University of Florida, Gainesville, FL, USA

Supplemental Table 1. Comparison of Sarnat score, max nSOFA, and combined models for association with adverse Weeke outcome

Model performance

Model	Predictors	AUROC	95% CI	ΔAUROC vs Sarnat alone	ΔAUROC vs nSOFA alone	Correctly classified at 0.5 cutoff	IDI vs Sarnat alone	IDI vs nSOFA alone
Model 1	Sarnat score alone	0.658	0.551–0.766	Reference	—	86/122 (70.5%)	Reference	—
Model 2	max nSOFA alone	0.633	0.520–0.747	−0.025	Reference	89/122 (73.0%)	—	Reference
Model 3	Sarnat score + max nSOFA	0.693	0.588–0.799	+0.035	+0.060	89/122 (73.0%)	+0.029	+0.036

Classification details at predicted probability cutoff of 0.5

Model	Predictors	True negatives	False positives	False negatives	True positives	Correctly classified
Model 1	Sarnat score alone	86	0	36	0	86/122 (70.5%)
Model 2	max nSOFA alone	79	7	26	10	89/122 (73.0%)
Model 3	Sarnat score + max nSOFA	82	4	29	7	89/122 (73.0%)

AUROC indicates area under the receiver operating characteristic curve; IDI, integrated discrimination improvement; nSOFA, neonatal Sequential Organ Failure Assessment. Correct classification was calculated using a predicted probability cutoff of 0.5. IDI was calculated from the difference in discrimination slopes between models using row-level predicted probabilities.

Supplementary Table 2. Multivariable Logistic Regression Model for Weeke

**Variable**	**Beta**	**Beta 95% CI**	**OR**	**OR 95% CI**	**p-value**
Intercept	-5.682	-8.156 to -3.624	-	-	<0.001
lactate_max	-0.016	-0.170 to 0.135	0.98	0.84 to 1.14	0.839
gas_base_delta_min	-0.170	-0.327 to -0.029	0.84	0.72 to 0.97	0.018
pt_max	0.134	0.043 to 0.236	1.14	1.04 to 1.27	0.003

Supplemental Table 3. Clinical characteristics among patients with and without a Bayley assessment.

Infant characteristics	Bayley not done (n=56)	Bayley done (n=66)	p-value
Male, n (%)	31 (56%)	33 (50%)	0.58
Gestational age, weeks, mean ± SD	39 ± 2	38 ± 2	0.31
Birthweight, grams, mean ± SD	3426 ± 661	3477 ± 712	0.68
C-section delivery, n (%)	34 (62%)	38 (58%)	0.71
Non-reassuring fetal heart tones, n (%)	23 (42%)	20 (30%)	0.25
Sentinel event*, n (%)	25 (45%)	24 (36%)	0.09
Apgar score 1 min, median (IQR)	2 (1, 2)	1 (1, 3)	0.38
Apgar score 5 min, median (IQR)	5 (3, 6)	5 (2, 7)	0.45
Apgar score 10 min, median (IQR)	6 (4, 7)	5 (3, 7)	0.09
Sarnat score II, n (%)	39 (71%)	31 (47%)	0.01
Sarnat score III, n (%)	14 (25%)	15 (23%)	0.83
Length of stay, median (IQR)	12 (8, 23)	11 (8, 21)	0.62
Intubation, n (%)	35 (64%)	33 (50%)	0.2
Vasoactive inotropic medications, n (%)	12 (22%)	14 (21%)	0.99
Antiepileptic drug use, n (%)	21 (38%)	16 (24%)	0.12
Arterial cord pH, median (IQR)	7.02 (6.91, 7.15)	7.06 (6.95, 7.14)	0.39
Arterial cord base deficit, mean ± SD	-13.7 ± 5.4	-12.5 ± 6.2	0.37
Maximum nSOFA, median (IQR)	0 (0, 3)	0 (0, 4)	0.50

Supplementary Table 4. Multivariable Logistic Regression Model for Bayley

Variable	Beta	Beta 95% CI	OR	OR 95% CI	p-value
Intercept	-2.397	-4.004 to -0.999	-	-	<0.001
gas_base_delta_min	0.046	-0.113 to 0.210	1.05	0.89 to 1.23	0.563
lactate_max	0.217	0.035 to 0.417	1.24	1.04 to 1.52	0.019
nsofa_max_0_12	0.128	-0.061 to 0.329	1.14	0.94 to 1.39	0.184

Missing data were handled using complete-case analysis. For the Weeke model, 118 of 122 observations were included (4 excluded due to missing max PT value). For the Bayley model, all 66 observations were included.

Supplemental Figure 1. Significant maximum and minimum laboratory values between Weeke groups. Normal Weeke score <5. Abnormal Weeke score ≥5. Absolute values shown with median and interquartile range or mean and standard deviation. For some laboratory types, only one measure from birth to 12 hours may have occurred and values may be present in both maximum and minimum comparisons. ns – not significant. * - p<0.05. ** - p<0.01. *** - p<0.001. **** - p<0.0001.

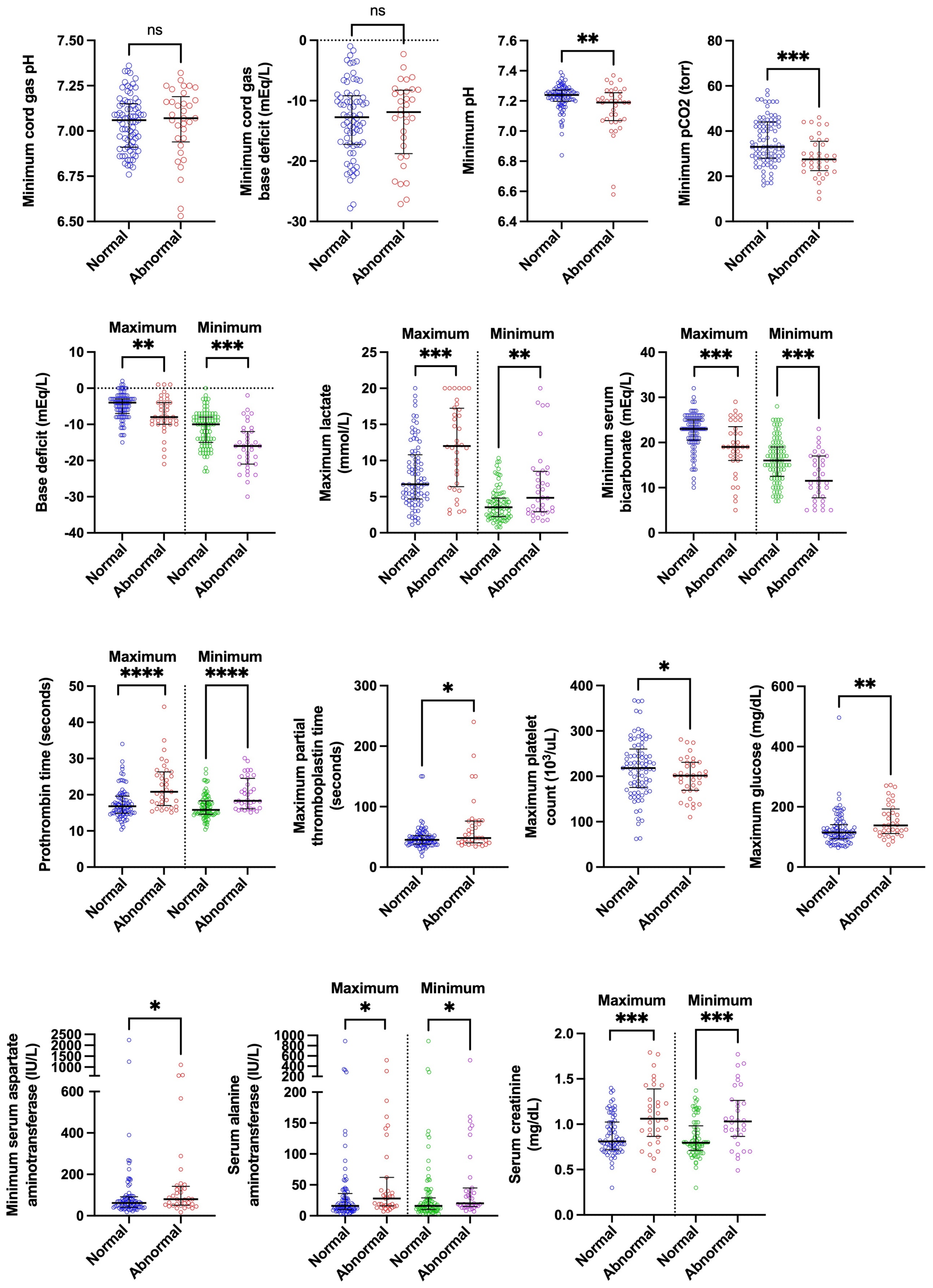

Supplemental Figure 2. Significant maximum and minimum laboratory values between NDI groups. NDI – neurodevelopmental impairment (any composite Bayley score <85 in any domain). Absolute values shown with median and interquartile range. ns – not significant. * - p<0.05. ** - p<0.01.

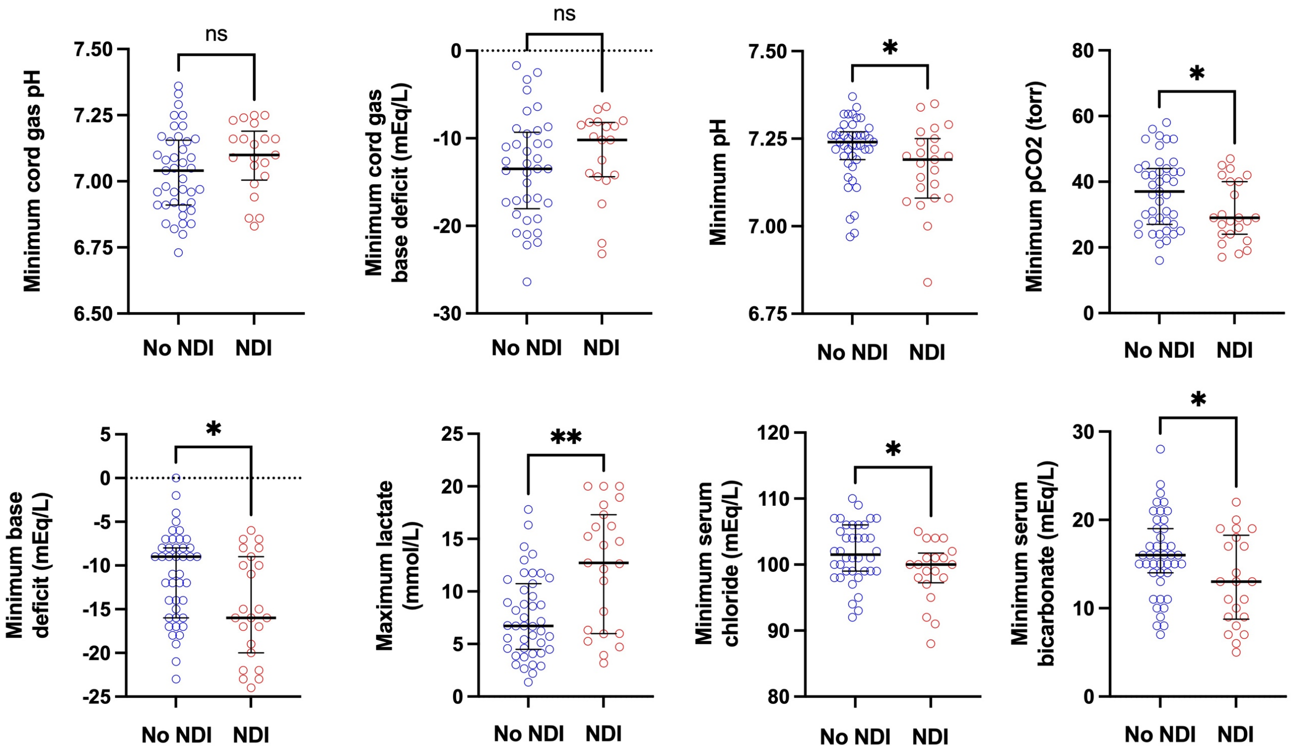

Supplemental Figure 3. Probability tree for abnormal Weeke score. *Lactate* – Maximum lactate up to 12 hours after birth. *Apgar* – 5-minute Apgar score. *Sentinel* – presence of a sentinel event. *Overall risk* – frequency of an abnormal Weeke MRI score (≥5). Color coding – green (low risk) to red (high risk).

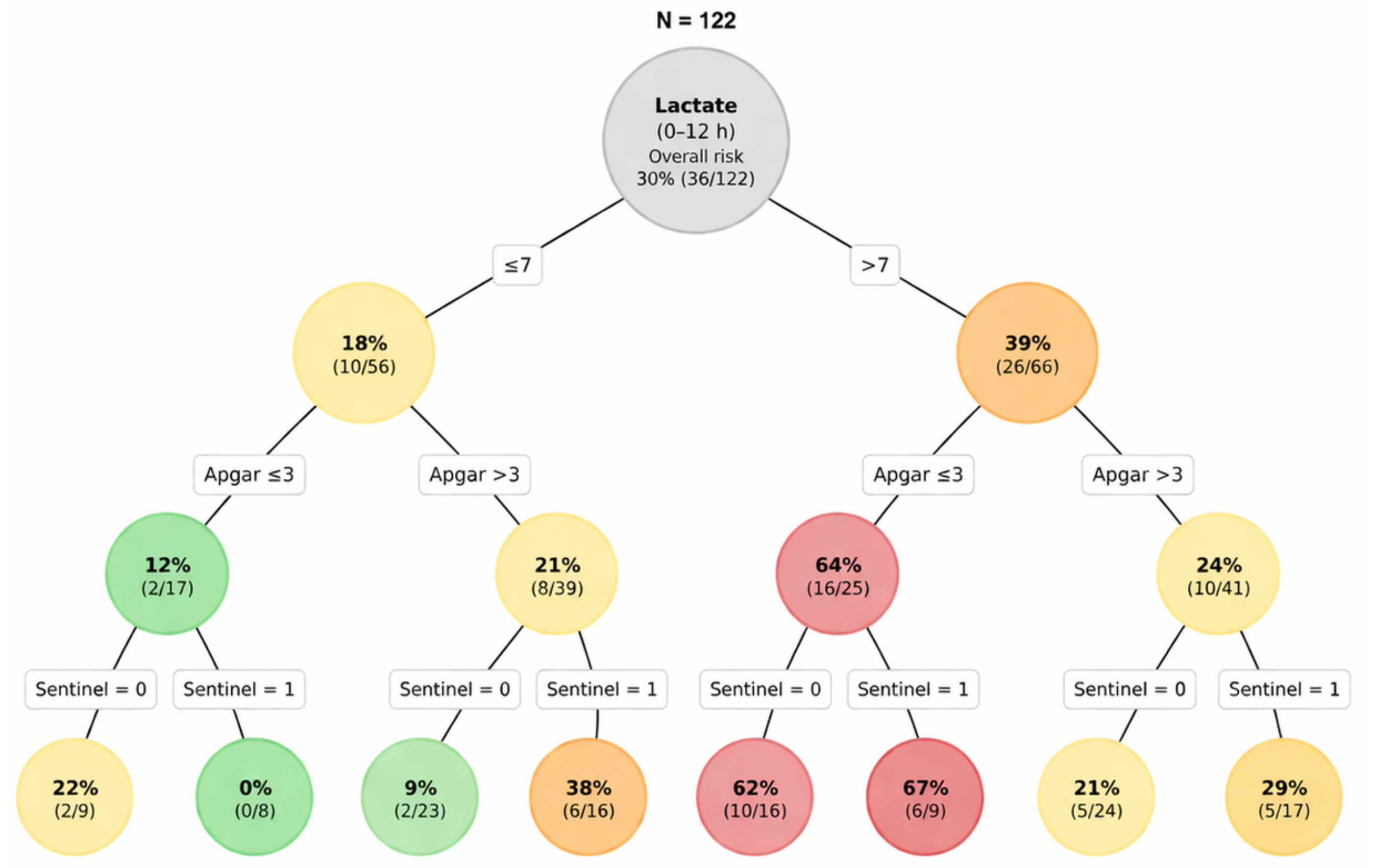

Supplemental Figure 4. Probability tree for neurodevelopmental impairment (NDI). NDI was any Bayley III composite score <85. *Lactate* – Maximum lactate up to 12 hours after birth. nSOFA – maximum neonatal sequential organ failure assessment score up to 12 hours after birth. Color coding – green (low risk) to red (high risk).

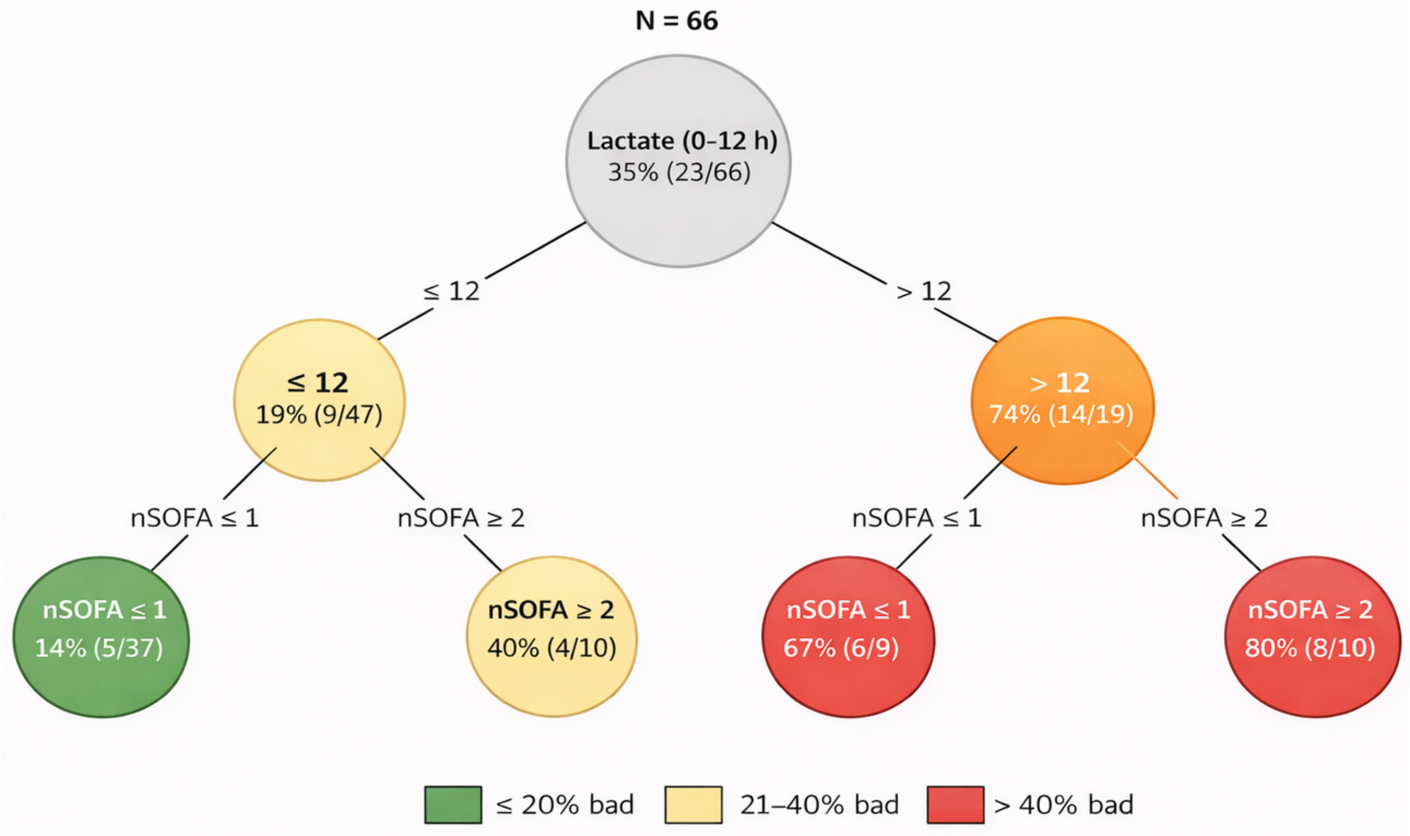

Supplement: Supplementary file 1 [file Datasheet1.pdf]
